# Enhancing reach and effectiveness of recruitment through email: a study within a trial (SWAT)

**DOI:** 10.1186/s13063-026-09902-1

**Published:** 2026-08-06

**Authors:** Theresa E. Erwig, Aaron A. Call, Nathan F. Dieckmann, Mark A. Reger, Alan R. Teo

**Affiliations:** 1 VA Portland Health Care System, Center to Improve Veteran Involvement in Care (CIVIC), 3710 SW US Veterans Hospital Road (R&D 66), Portland, OR 97239 USA; 2 Division of Psychology, School of Medicine, Oregon Health & Science University, 3266 SW Research Dr, 4th Floor, Portland, OR 97239 USA; 3 School of Nursing, Oregon Health & Science University, 3455 SW US Veterans Hospital Rd., Portland, OR 97239 USA; 4 School of Public Health, Oregon Health & Science University-Portland State University, 1850 SW 5th Ave, Portland, OR 97201 USA; 5 VA Puget Sound Health Care System, 1660 S Columbian Way, Seattle, WA 98108 USA; 6 Department of Psychiatry and Behavioral Sciences, University of Washington, 1959 NE Pacific Street, Box 356560, Seattle, WA 98195 USA; 7 Department of Psychiatry, Oregon Health & Science University, 3181 Sam Jackson Park Rd, Portland, OR 97239 USA

**Keywords:** Online recruitment, Recruitment strategies, Health message framing, Prenotification, Veterans health research, Caring contacts

## Abstract

**Background:**

Best practices for efficient and effective email recruitment are unclear. This study within a trial (SWAT) aimed to evaluate the effect of an email’s subject line and a prenotification email, as well as explore the impact of sociodemographic characteristics, on recruitment.

**Methods:**

The SWAT was embedded within the Buddy Check Postcard Project (BCPP) trial, which investigated sending brief caring postcards to older veterans to reduce loneliness. The SWAT was a quasi-experimental study (*n* = 1945) with time-based allocation that tested three subject lines and use of a prenotification (email notifying participants to expect a study invitation). Subject lines each emphasized a different potential motivator for research participation: benefit to self (incentive), benefit to others (altruism), and official Department of Veterans Affairs (VA) email (institutional reference). The primary outcome was reach (whether recipients opened the email) and the secondary outcome was effectiveness (whether recipients completed the baseline survey). Sociodemographic characteristics considered were age, sex assigned at birth, race, ethnicity, and rurality.

**Results:**

The institutional subject line was associated with higher odds of opening the email compared to the altruism (adjusted odds ratio (AOR) = 1.93, 95% confidence interval (CI): [1.46, 2.55]) and incentive subject lines (AOR = 1.54, 95% CI: [1.19, 1.99]), adjusting for sociodemographic characteristics, site, and prenotification. The institutional subject line was also associated with higher odds of baseline survey completion than the altruism subject line (AOR = 2.44, 95% CI: [1.08, 5.50]), but not compared to the incentive subject line (AOR = 1.42, 95% CI: [0.73, 2.76]). Receiving a prenotification email was not significantly associated with increased reach or effectiveness. Non-Hispanic/Latino White urban participants were more likely to open the recruitment email. Younger, female, non-Hispanic/Latino White participants were more likely to complete the baseline survey. There was no evidence of sociodemographic characteristics moderating the effects of either the subject line or prenotification.

**Conclusions:**

Veterans who received an email subject line with an institutional reference to the VA were more likely to open the email and enroll in a clinical trial than veterans who received an incentive or altruistic subject line. This study did not detect clear evidence of a substantial benefit for recruitment to sending prenotification emails.

**Supplementary Information:**

The online version contains supplementary material available at https://doi.org/10.1186/s13063-026-09902-1.

## Background

Achieving representative recruitment targets in randomized controlled trials (RCTs) is a consistent and pervasive challenge in health research [1–4]. Poor participant recruitment is the most common reason for premature discontinuation of RCTs [5], and just 56% of RCTs are estimated to successfully reach their targeted sample size [6]. Veterans’ geographic mobility, survey fatigue, and stigma associated with mental health issues may provide further challenges for researchers recruiting them as study participants in large-scale studies [7]. Recruitment bias is of particular concern to veteran populations, where researchers have noted difficulties recruiting racially and ethnically diverse veterans and female and lesbian, gay, bisexual, transgender, queer or questioning, or other gender identities or sexual orientation (LGBTQ +) veterans, leading to underrepresented populations being excluded from research [8–11].

Email recruitment may be a promising approach to improve health research recruitment due to its broad accessibility, efficiency, and low cost [12]. Email may be particularly effective at reaching the veteran population because of the inclusion of veterans’ emails in their electronic medical records and use of secure messaging. In a trial enrolling older military veterans, response rates were four times higher when individuals were contacted through email compared to postal mail [13]. However, these same traits that make email appealing for recruitment for research studies have also led to significant oversaturation among potential participants [14]. “Email fatigue” or “email overload” is common among potential research participants, and this may decrease response rates or lead to significant non-response error [15–17]. Furthermore, digital recruitment techniques may result in unrepresentative study samples with younger, more affluent, and healthier populations often being overrepresented [4].

Two specific elements that may affect whether prospective participants open, read, and respond to an email are the subject line of the email and whether recipients are expecting to receive a study invitation (prenotification).

### Email subject line

Email subject lines are a primary determinant of whether an email is opened and read, functioning as a filtering mechanism for recipients [12, 18]. Subject lines offer an opportunity to frame study invitations, leveraging “framing effects” wherein decision-making is influenced by how a situation is presented [19]. While informative and direct subject lines are generally preferred [20], the impact of different framing strategies in research recruitment is evolving. Early studies on email surveys sometimes found high response rates with blank subject lines [21], but more recent evidence suggests that concise, action-oriented subject lines are most effective for health-related communications, with additional verbiage potentially reducing email open rates [12, 22].

Studies have had mixed results when looking at the impact of including an incentive versus an altruistic appeal in email subject lines. Some studies have found that including a request or plea for help [23, 24] or using egoistic framing or mentioning an incentive [25, 26] increased response rate, while others found no impact [27]. The results of a 2024 study of email recruitment of businesses to an economic survey suggested that including a motivation-based appeal (either altruistic or self-interested) actually reduced response rates compared to a simplified subject line that only stated that a particular institution was reaching out for the business to participate in an economic survey [14].

There is some preliminary evidence that incorporating an appeal to an official institutional authority in an email subject line may enhance response rates. For instance, one study that tested a subject line that referenced a request from a university’s vice president found that this subject line significantly increased response rates to a web survey [28]. Other studies considering sender title found that direct mail authored by a state official resulted in greater organ donation registration rates [29] and marketing emails sent from a company’s CEO had higher email open rates [30]. However, including university affiliation in the subject line did not improve email response rates in two other trials [27], and another study found that incorporating the institution and title of the sender into the body of an email did not enhance response rates [24]. Recipients’ characteristics, including level of autonomy, may impact how they may respond to such messages [31], and message type may affect how recipients respond to a particular expert or institution [32]. These contradictory results suggest that email subject lines can play an important role in recruitment, but it is still unclear what type of email subject line is the most effective.

### Prenotification emails

Prenotification is a common tool in recruitment, and it involves contacting a prospective participant prior to sending detailed recruitment materials [33–35]. There is some preliminary evidence that suggests that email delivery of a prenotification may have greater effects than postal delivery [36]. However, studies on using email prenotifications to increase response rates have mixed results [36–41]. Moreover, prenotifications have not been thoroughly studied among veteran populations and underrepresented populations historically excluded from research.

### The present study

The overarching goal of the present study was to examine the effects of changing the recruitment email’s subject line and sending a prenotification on recruitment outcomes. We also explored the impact of sociodemographic characteristics, including age, sex assigned at birth, race, ethnicity, and rurality, on how participants responded to recruitment emails. Drawing from existing literature, this study focused on the framing of subject lines that emphasized three unique potential motivators for research participation: benefit to self (incentive), benefit to others (altruism), and an institutional reference. This study also evaluated the effects of a prenotification email sent before the study invitation email.

## Methods

### Design

A quasi-experimental SWAT with time-based allocation was embedded in the Buddy Check Postcard Project (BCPP) RCT (ClinicalTrials.gov ID: NCT05484297). BCPP is a multi-site, pragmatic RCT funded by the VA investigating whether sending brief caring postcards signed by peer specialists to older Veterans with limited engagement in VA care decreases loneliness (primary outcome) and improves treatment engagement and reduces suicide and all-cause mortality (secondary outcomes).

We embedded this SWAT during BCPP’s initial recruitment, and then preliminary SWAT findings informed further recruitment efforts. The VA Central Institutional Review Board approved BCPP with multiple email recruitment methods that this SWAT tested. The SWAT adheres to the SWAT Reporting Guidelines [42] checklist (Additional file 1).

### Participants

All participants in the SWAT were identified as potentially eligible participants for the BCPP trial, which acted as the host trial. BCPP trial participants were VA patients from three sites across the USA who were ≥ 50 years old (the cutoff was initially ≥ 60 years old until 5/22/2023), screened positive for loneliness via the 3-item UCLA Loneliness Scale [43], had at least four appointments in the prior 6–18 months in primary care or mental health, and either had zero completed or scheduled appointments in the past 6 months or were in the 90th percentile or higher in terms of no-shows or appointments canceled by patient in the prior 12 months. In addition, to be eligible for the SWAT, participants had to have an email address on file. Those with emails that were not delivered (e.g., bounced emails) or who were initially contacted via postal mail were excluded. Participants were blind to their participation in this SWAT. We collected data from Qualtrics, which captures email and survey completion data, and VA’s Corporate Data Warehouse (CDW), which contains administrative health record data.

### Interventions

This SWAT explored two different interventions: recruitment email subject line and prenotification email. Table 1 details the subject lines sent and time-based allocations among intervention groups.

**Table 1:** Recruitment email subject lines and prenotification emails as sent to participants

Framing	Recruitment email subject line	Prenotification email	Run dates	Sender email domain	Participants emailed (*n*)
Incentive	“ < First name >, participate in the Buddy Check Postcard Project and earn $100”	Not sent	4/4/23–6/5/23	@qemailserver.com	471
Altruism	“ < First name >, want to help other Veterans like you?”	Not sent	6/12/23–8/7/23	@qemailserver.com (6/12/23–6/25/23) @qualtrics-research.com (6/26/23–8/7/23)	354
Institutional	“ < First name >, VA Research Study Invitation [Official]”	Not sent	8/14/23–9/11/23, 9/16/24–10/11/24, 10/28/24–11/1/24, 11/18/24–11/29/24	@qualtrics-research.com	499
Institutional	“ < First name >, VA Research Study Invitation [Official]”	Sent	9/25/23–4/4/24	@qualtrics-research.com	621

Recruitment emails were sent in batches once per week (on Mondays 4/4/23–9/11/23, then on Tuesdays 9/18/23–11/29/24) from either an @qemailserver.com (4/4/23–6/25/23) or an @qualtrics-research.com (6/26/23–4/4/24) email address and included a brief overview of the study as well as options to respond by starting an eligibility screener, reading more about the study, or declining participation. The content of recruitment emails changed slightly during the recruitment period (Additional file 2). Prenotification emails were sent in batches on Mondays from an @va.gov email address, were sent with an institutional subject line, included a similar overview of the study, let veterans know to be on the lookout for an invitation, and did not provide response options (Additional file 3).

### Outcomes

The primary outcome was reach of the recruitment email. We operationalized reach as whether participants opened the initial recruitment email. Qualtrics automatically captured data on whether each email was opened based on whether a tiny transparent pixel was loaded by email recipients.

The secondary outcome was effectiveness of recruitment. We assessed effectiveness by tracking whether participants completed the BCPP baseline surveys within 10 days of when the initial recruitment email was sent. We used this 10-day limit because additional recruitment efforts (text message reminders and phone calls) were made after 10 days. An additional reminder email with the same subject line as the initial recruitment email was sent to participants who had not already responded 5 days after the initial recruitment email. Participants could access the baseline survey after providing their contact information, consenting to the host trial, and completing the loneliness screener.

### Sample size

The SWAT sample size (*n* = 1945) depended on the host BCPP trial; therefore, no formal a priori sample size calculation was performed, which is in line with SWAT methodology [42]. Capacity of the research team to implement the changes in subject line and prenotification emails determined the run dates for each intervention group. We stopped the SWAT after 1 year of recruitment to implement preliminary findings (*n* = 1589). Thereafter, a technical error with our email server resulted in an additional batch of participants (*n* = 356) not receiving a prenotification email.

### Statistical methods

We summarized baseline participant data and the outcome measures using frequency (%) or mean with standard deviation (SD) as appropriate.

Logistic regression models compared the primary and secondary outcomes between recruitment email subject line groups and the prenotification and no prenotification groups, adjusting for site and sociodemographic characteristics (age, sex assigned at birth, race/ethnicity, and rurality). Due to low prevalence of ethnic and racial minorities in the sample, we combined data into three categories: non-Hispanic/Latino White, non-White and/or Hispanic/Latino, and Unknown. No race/ethnicity data was missing, and any participants for whom race or ethnicity was unknown were handled as an explicit analytic category. Age was treated as a continuous variable for reports of odds ratios and a categorical variable, by decade, for presenting predicted probabilities. We used odds ratios (ORs) and 95% confidence interval (CI), with significance levels set at *p* < 0.05 with two-tailed tests, for comparisons and present predicted probabilities as point estimate for ease of interpretation.

Two sensitivity analyses evaluated the robustness of the primary findings. First, given the potential for sender email domain to confound associations between email subject line and recruitment outcomes, sender email domain was added as an additional predictor variable to the primary logistic regression models and changes in email subject line-related outcomes were analyzed. Second, we considered an alternative effectiveness definition of baseline survey completion within 5 days of the initial email invitation (before distribution of the reminder email), rather than within 10 days as specified in the primary analyses, and re-estimated logistic regression models to assess the consistency of associations for subject line condition and prenotification email receipt.

To assess whether any sociodemographic characteristics of participants moderated the relationship between recruitment email subject line or prenotification email and reach, we ran separate adjusted logistic regression analyses with an interaction term between recruitment email subject line or prenotification and each sociodemographic characteristic (age, sex assigned at birth, race/ethnicity, and rurality). The Benjamini–Hochberg procedure was used to control the false discovery rate for these exploratory tests [44]. Due to substantially reduced power, we did not analyze moderation effects for effectiveness. All analyses were conducted using Stata version 18 (Stata Corp.).

## Results

### Participant flow and baseline data

**Fig. 1:**
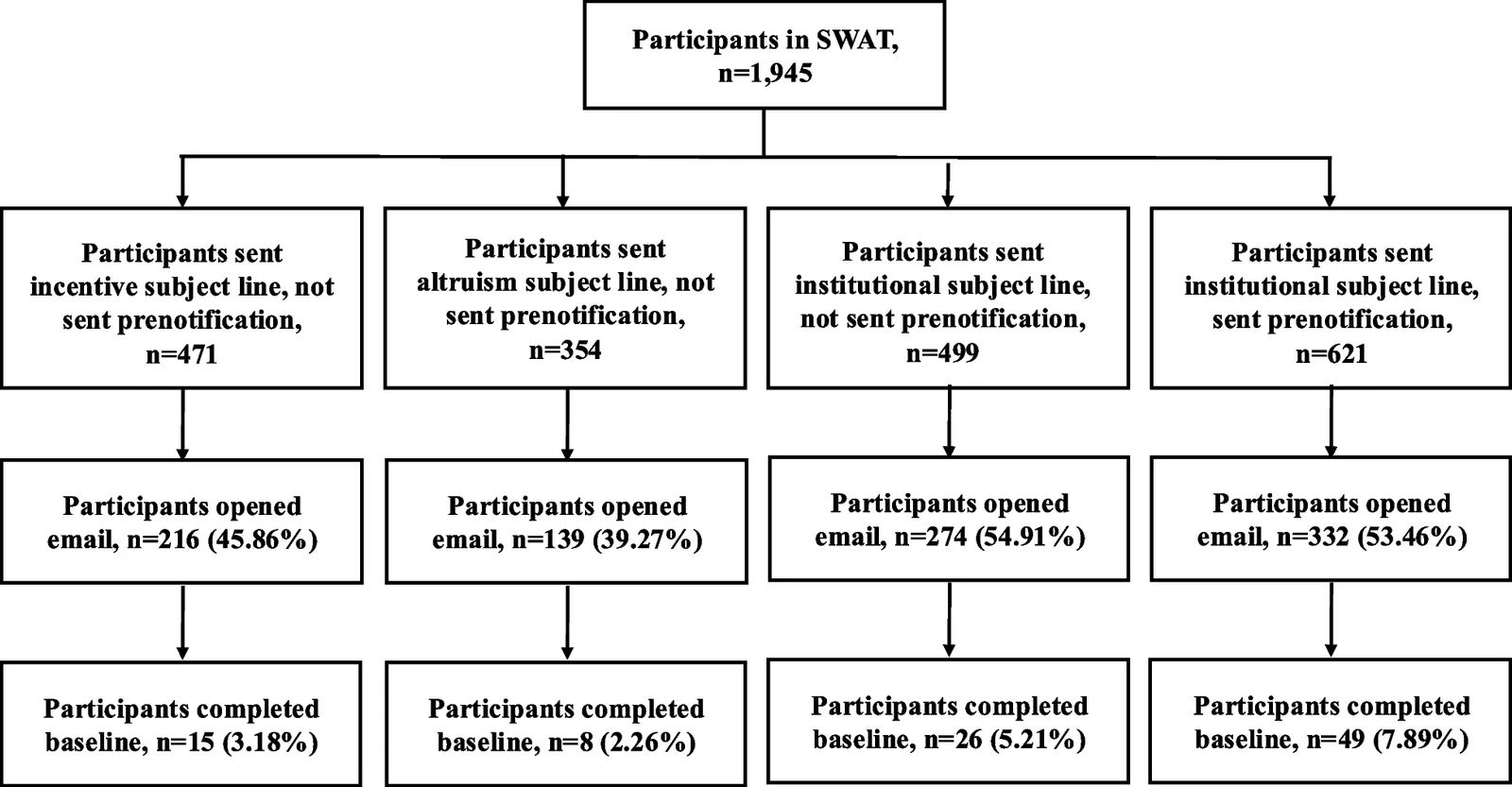
Participant flow diagram

Figure 1 provides the observed SWAT participant flow and recruitment outcomes across intervention groups.

Table 2 provides baseline characteristics of the sample in the SWAT. The mean age was 68.1 years, with a standard deviation of 9.9 years. Males comprised 91% of the sample, and 74% were white and 89% were non-Hispanic or Latino. Nearly two-thirds of the sample was urban with the remaining one-third being rural.

**Table 2:** Sociodemographic characteristics of the sample (*n* = 1945)

	**Mean or frequency**	**Standard deviation or %**
**Age**	68.06	± 9.93
**Sex assigned at birth**		
Male	1,771	(91.05%)
Female	174	(8.95%)
**Race**^a^		
American Indian or Alaska Native	17	(0.87%)
Asian	34	(1.75%)
Black or African American	199	(10.23%)
Native Hawaiian or Other Pacific Islander	23	(1.18%)
White	1,434	(73.73%)
Two or more races	12^b^	(0.62%)
Unknown	226	(11.62%)
**Ethnicity**		
Hispanic or Latino	53	(2.72%)
Not Hispanic or Latino	1,735	(89.20%)
Unknown^c^	157	(8.07%)
**Rurality**^d^		
Urban	1,273	(65.45%)
Rural	672	(34.55%)

^a^Descriptive statistics were provided for race and ethnicity despite insufficient sample size in certain subgroups for detailed inclusion in further data analysis [45]

^b^These included 1 individual who was American Indian or Alaska Native and Black or African American; 4 individuals who were American Indian or Alaska Native and White; 2 individuals who were American Indian or Alaska Native, Black or African American, and White; 2 individuals who were Black or African American and White; and 3 individuals who were Native Hawaiian or Other Pacific Islander and White

^c^Unknown ethnicity includes declined to answer, unknown, and unknown by patient

^d^Rurality was determined based on Rural–Urban Commuting Area codes (RUCA) [46]. Rural includes highly rural and insular islands

### Primary outcome: opening of recruitment email (reach)

Participants who received a recruitment email with the institutional subject line were more likely to open the email, compared to those who received a recruitment email with either the altruism or incentive subject lines. Specifically, receiving the institutional subject line was associated with 1.93 times higher odds of opening the recruitment email compared to recipients of the altruism subject line (AOR = 1.93, 95% CI: [1.46, 2.55], *p* < 0.001) and 1.54 times higher odds compared to recipients of the incentive subject line (AOR = 1.54, 95% CI: [1.19, 1.99], *p* = 0.001). Recipients of the institutional subject line were predicted to open the recruitment email 54.68% of the time, whereas recipients of the altruism subject line were only predicted to open the recruitment email 38.79% of the time, and recipients of the incentive subject line were predicted to open the recruitment email 44.92% of the time (Table 3).

**Table 3:** Reach and effectiveness of recruitment email based on subject line, prenotification email, and sociodemographics (*n* = 1945)

	**Observed opening email *n* (%)**	**Adjusted predicted probability of opening email [95% CI]**	**Observed baseline survey completion *n* (%)**	**Adjusted predicted probability of baseline survey completion [95% CI]**
**Recruitment email subject line**				
Institutional	606 (54.11%)	54.68% [51.46, 57.89]	75 (6.70%)	5.87% [4.45, 7.28]
Altruism	139 (39.27%)	38.79% [33.45, 44.13]	8 (2.26%)	2.58% [0.76, 4.40]
Incentive	216 (45.86%)	44.92% [40.03, 49.81]	15 (3.18%)	4.29% [2.01, 6.57]
**Prenotification email**				
Not sent	629 (47.51%)	49.55% [46.67, 52.42]	49 (3.70%)	4.21% [3.00, 5.42]
Sent	332 (53.46%)	49.12% [44.61, 53.62]	49 (7.89%)	6.32% [4.37, 8.28]
**Age**				
50–59	227 (51.95%)	52.44% [47.69, 57.20]	39 (8.92%)	8.30% [5.72, 10.88]
60–69	278 (45.87%)	46.61% [42.65, 50.58]	35 (5.78%)	5.86% [3.96, 7.75]
70–79	349 (50.29%)	49.71% [45.99, 53.44]	19 (2.74%)	2.88% [1.60, 4.15]
80 +	107 (51.44%)	50.18% [43.44, 56.92]	5 (2.40%)	2.39% [0.33, 4.44]
**Sex**				
Male	875 (49.41%)	49.37% [47.08, 51.67]	75 (4.23%)	4.36% [3.41, 5.32]
Female	86 (49.43%)	49.79% [42.32, 57.25]	23 (13.22%)	10.33% [6.27, 14.38]
**Race/ethnicity**				
Non-Hispanic White	711 (50.86%)	50.95% [48.34, 53.55]	82 (5.87%)	6.04% [4.80, 7.29]
Non-White or Hispanic	134 (41.74%)	41.59% [36.11, 47.06]	7 (2.18%)	2.14% [0.57, 3.71]
Unknown	116 (51.33%)	51.03% [44.57, 57.50]	9 (3.98%)	3.45% [1.25, 5.64]
**Rurality**				
Urban	641 (50.35%)	51.11% [48.39, 53.83]	58 (4.56%)	4.71% [3.55, 5.88]
Rural	320 (47.62%)	46.21% [42.47, 49.95]	40 (5.95%)	5.60% [3.94, 7.26]

Receiving a prenotification email before a study invitation was not associated with different odds of opening the study invitation email (AOR = 0.99, 95% CI: [0.78, 1.25], *p* = 0.911). Regardless of whether participants were sent a prenotification email, they were predicted to open the study invitation email 49.12–49.55% of the time (Table 3).

Being non-Hispanic/Latino White and urban was significantly associated with more email reach, controlling for VA site, email subject line, and prenotification emails. No other sociodemographic characteristics significantly predicted reach. Non-White and/or Hispanic/Latino participants had 33% lower odds of opening the recruitment email compared to non-Hispanic/Latino White participants (AOR = 0.67; 95% CI:[0.52, 0.87], *p* = 0.002). Being non-Hispanic/Latino White predicted a 50.95% probability of opening the email, while being non-White and/or Hispanic/Latino predicted a 41.59% probability of opening the email (Table 3). Rural participants had 18% lower odds of opening the recruitment email compared to urban participants (AOR = 0.82; 95% CI:[0.68, 1.00], *p* = 0.046). Being urban predicted a 51.11% probability of opening the email, while being rural predicted a 46.21% probability of opening the email.

### Secondary outcome: completion of baseline survey (effectiveness)

The institutional subject line was associated with higher effectiveness compared to the altruism subject line, but its effectiveness was not statistically significant compared to the incentive subject line. Receiving a recruitment email with the institutional subject line was associated with 2.44 times higher odds of completing the baseline survey within 10 days, compared to those who received a recruitment email with the altruism subject line (AOR = 2.44, 95% CI:[1.08, 5.50], *p* = 0.032). Participants who received a recruitment email with the institutional subject line did not have significantly different odds of completing the baseline survey within 10 days, compared to those who received a recruitment email with the incentive subject line (AOR = 1.42, 95% CI:[0.73, 2.76], *p* = 0.303). Recipients of the institutional subject line were predicted to complete the baseline survey within 10 days 5.87% of the time, whereas recipients of the altruism subject line were only predicted to complete the baseline survey within 10 days 2.58% of the time and recipients of the incentive subject line were only predicted to complete the baseline survey within 10 days 4.29% of the time (Table 3).

Receiving a prenotification email before a study invitation was not significantly associated with effectiveness in terms of odds of completing the baseline survey within 10 days of receiving the study invitation email (AOR = 1.56, 95% CI: [0.95, 2.58], *p* = 0.082). Receiving a prenotification email predicted a 6.32% probability of completing the baseline survey within 10 days, while not receiving a prenotification email predicted a 4.21% probability of completing the baseline survey within 10 days (Table 3).

Being younger, female, and non-Hispanic/Latino White was significantly associated with more effectiveness, controlling for VA site, email subject line, and prenotification emails. Rurality was not associated with a difference in effectiveness (Table 3). When controlling for email subject line, prenotification email, site, and other sociodemographic characteristics, each additional year of age was associated with 5% lower odds of completing the baseline survey within 10 days (AOR = 0.95; 95% CI: [0.93, 0.98], *p* < 0.001). Female participants had 2.72 times higher odds of completing the baseline survey within 10 days compared to male participants (AOR = 2.72, 95% CI: [1.63, 4.55], *p* < 0.001). Being female predicted an 10.33% probability of completing the baseline survey within 10 days, while being male predicted a 4.36% probability of completing the baseline survey within 10 days. Participants identified as non-White and/or Hispanic/Latino had 68% lower odds of completing the baseline survey within 10 days compared to non-Hispanic/Latino White participants (AOR = 0.32; 95% CI:[0.15, 0.72], *p* = 0.006). Being non-Hispanic/Latino White predicted a 6.04% probability of completing the baseline survey within 10 days, while being non-White and/or Hispanic/Latino predicted a 2.14% probability of completing the baseline survey within 10 days.

### Moderating effects of sociodemographic characteristics on the impact of subject line or prenotification on reach

No evidence suggested that sociodemographic characteristics significantly moderated the relationship between recruitment email subject line or prenotification email and reach, after controlling the false discovery rate.

### Sensitivity analyses

In sensitivity analyses that additionally adjusted for sender email domain, results were generally consistent with the primary models, with the institutional subject line remaining the highest-performing condition for email opening and baseline survey completion; however, the relative ordering of the altruism and incentive subject lines changed, such that incentive showed relatively lower odds of email opening and baseline survey completion (Additional file 4).

In sensitivity analyses defining baseline survey completion as completion within 5 days of the initial email invitation, subject line effects were similar to those observed in the primary analyses. Receipt of the prenotification email was associated with higher odds of baseline survey completion and reached statistical significance under this alternative outcome definition.

## Discussion

This SWAT supported the hypothesis that a brief, direct email subject line improves research recruitment. More specifically, our results suggest that the subject line that was associated with the most reach among invited veterans was institutional, followed by incentive, and lastly altruism. The institutional subject line also outperformed altruism in terms of its association with increased effectiveness, although its outperformance vis-à-vis incentive was not statistically significant for effectiveness. While promising in terms of the potential effects of framing subject lines to boost recruitment, the quasi-experimental design and time-based allocation of subject lines in this SWAT suggest that further research with randomized allocation is needed to confirm these findings.

The relative success of the institutional subject line in this SWAT may be explained either by prior literature [12, 14, 20–26, 28] on the use of institutional authority and perhaps the specific population recruited or by prior literature on the benefit of a straightforward, informative subject line [20, 22]. Regarding the former potential explanation, previous studies have suggested recipients’ characteristics may impact how they may respond to institutions [31]. Given that all SWAT participants were veterans, we speculate that their veteran identity impacted their responsiveness to the institutional subject line, given the military’s culture of respect and comfort with VA communication. Military culture’s emphasis on compliance, following orders, and chain of command may impact veterans’ mindset when engaging in research recruitment efforts [47]. Participants’ veteran status may mediate how they respond to an institutional frame that appeals to a veteran institution. This may help to explain why the results differ from some previous non-veteran studies that found appealing to an institutional authority figure in an email subject line did not impact responsiveness [24, 27], and bolsters the cumulative evidence that the specific identity of participants should be considered when comparing the results of multiple email recruitment studies.

Alternatively, perhaps the straightforward and informative nature of the institutional subject line explains its relative success in this study. As compared to the institutional subject line, the altruism and incentive subject lines were a bit less informative since they did not explicitly state that the email was an invitation to participate in a research study nor disclose the sponsor of the study. This SWAT may then actually reinforce previous literature that suggests that subject lines should be “informative and straightforward” and should clearly reference the purpose and sponsor of the survey or study [20] and that an “action only” subject line may perform better than those including additional potential motivators [22]. This SWAT therefore adds to the cumulative evidence supporting a clear, concise, and informative subject line.

Moreover, the findings regarding the impact of email subject line framing assume that the content of the email subject lines elicited the intended framing effects of institutional, incentive, and altruism. Although the framing categories have face validity to describe the subject lines used in the SWAT, further research could include focus groups or preliminary surveys about individuals’ perceptions of subject lines to confirm the framing is accurately characterized. It is also possible that subject lines do, in fact, elicit the intended framing when viewed in full, but some participants checked their email on a device that allowed them to see only part of the subject line (for example, they may not have seen the $100 at the end of the incentive subject line), which could have impacted the fidelity of the study. Further research could track how participants received emails, and whether they opened an email on phone or computer to determine the extent to which limited viewing of subject lines may impact results.

This SWAT did not detect clear evidence that sending a prenotification email substantially improved recruitment outcomes, which was consistent with previous cumulative research that suggested that prenotification emails had little to no effect [36–41]. Although statistically imprecise, it is possible that the 1.56 times higher odds of baseline survey completion associated with receiving the prenotification email may be clinically meaningful. Further research could consider whether the timing or content of a prenotification email impact its effects, or whether sending multiple prenotification emails could improve effectiveness. Tracking whether participants opened the prenotification email in future studies could also help explain its effects on study invitation reach and effectiveness. Additionally, considering the email sender of a prenotification email may also help explain its effects, since in the current study the prenotification email came from a different email address than the recruitment email. There may also have been a dilution effect in terms of effectiveness, since the effectiveness measure of baseline survey completion within 10 days included an additional reminder email 5 days after the initial recruitment email. In fact, there was some preliminary evidence of this dilution effect via the sensitivity analysis that considered an alternative effectiveness outcome of baseline survey completion within 5 days, where prenotification was then positively significantly associated with baseline survey completion.

Considering the recruitment effects of participants’ sociodemographic characteristics, this SWAT found that only race/ethnicity was associated with odds of opening the recruitment email, whereas race/ethnicity, age, and sex assigned at birth were associated with odds of completing the baseline survey within 10 days of receiving the recruitment email. Non-Hispanic/Latino White participants were more likely to open the recruitment email compared to non-White and/or Hispanic/Latino participants. Younger, female, and non-Hispanic/Latino White participants were more likely to complete the baseline survey within 10 days, compared to older, male, non-White and/or Hispanic/Latino participants. These findings have limited applicability because age was limited to over 50 in the host trial, and there was a general lack of diversity in terms of race and ethnicity, which led to the collapsing of these groups. This SWAT therefore lacks information about the nuanced differences between non-White and/or Hispanic/Latino groups [45], and used non-Hispanic White participants as the reference group, which could have led to inequitable interpretations [48]. In general, the impact of race/ethnicity on recruitment is well-documented in other research, and this SWAT’s findings that non-White and/or Hispanic/Latino participants were less likely to be recruited successfully into the RCT is consistent with prior research [10, 11]. This study does, however, hold promise for recruiting veterans assigned female at birth, as previous studies have noted that female veterans are generally underrepresented in health research [8, 9].

This SWAT found no evidence that supported that any specific sociodemographic characteristics moderated the reach of subject lines or prenotification emails. However, given limited statistical power for these analyses, these null findings should be interpreted cautiously and should not be interpreted as evidence that effects are uniform across populations. This was the first study we are aware of to conduct this analysis, and additional research is needed to confirm and replicate these findings. In addition, we did not test other sociodemographic characteristics, such as gender, education, income, geographic region of the country, language background, and country of origin. The general lack of diversity in our sample also limits the broader applicability of this finding.

Limitations include the non-randomized design, which could create a vulnerability to bias from unmeasured confounders, and the measurement of email opening, which could have resulted in some potential misclassification of the primary outcome. While we made efforts to maintain fidelity to our recruitment procedures (e.g., consistent research staffing, recruitment emails sent at the same day/time of the week), the varying time periods (months) across recruitment emails, secular trends, minor changes in email content, evolving operational workflows and increased study familiarity, and change in sender email domain name could have impacted outcomes. Specifically, the sender email domain changed from @qemailserver.com to @qualtrics-research.com during recruitment, such that emails with the incentive subject line were sent from the first email address, some of those with the altruism subject line were sent with both, and those with the institutional subject line were sent from the second email address. Given the overlap in sender domain epochs and subject line epochs, sender email domains may have confounded subject line results with plausible influence on trust and participant actions, despite sensitivity analyses. Further research may include a randomized design and may explicitly test the impact of sending emails via a more authoritative email domain. Moreover, we used Qualtrics’ metric of opened email for the primary reach outcome, which measured whether a tiny transparent pixel was loaded by email recipients. Although typically this occurs when a participant opens an email, it is possible to get false positives if a recipient preloads images or false negatives if a recipient’s security settings block automatic loading of images. Although still a useful signal to monitor trends, the potentially imprecise measurement of participants opening the recruitment email suggests that additional research should seek to replicate this SWAT’s findings using a different way of measuring reach. Because participants had to have an email address on file; results may not generalize to other veterans. Additionally, power was limited in some analyses (e.g., effectiveness, moderation analyses); further research should replicate these findings with a larger population.

## Conclusion

These findings highlight the importance of considering email subject lines in RCT design to enhance reach and effectiveness of recruitment. This study provides a blueprint for studying email techniques to maximize recruitment efforts, and our results support the use of institutional email subject lines over altruistic or incentive messages in recruiting veterans. This SWAT also calls into question whether using prenotification emails provides significant value to the recruitment process. Researchers should weigh any associated costs with the potential benefits of prenotifications.

## Supplementary Information

Additional file 1. SWAT reporting guideline checklist. Description of data: checklist for guidelines for reporting the results of a SWAT.

Additional file 2. Study invitation email. Description of data: versions of content of recruitment emails during SWAT recruitment.

Additional file 3. Prenotification email. Description of data: content of prenotification email.

Additional file 4. Supplemental sensitivity analyses. Description of data: stata output from supplemental sensitivity analyses.

## Data Availability

The data that support the findings of this study are available from the corresponding author upon reasonable request.

## Abbreviations

AOR: Adjusted odds ratios
CI: Confidence interval
BCPP: Buddy Check Postcard Project
RCT: Randomized control trial
SWAT: Study within a trial
VA: Department of Veteran Affairs
