# Supplementary material for: Enhancing reach and effectiveness of recruitment through email: a study within a trial (SWAT)
Source: Trials. 2026 Aug 6;27:643. doi: 10.1186/s13063-026-09902-1 (PMC13729685; doi:10.1186/s13063-026-09902-1)
Supplement: Supplementary file 1 [file 13063_2026_9902_MOESM2_ESM.pdf]

Additional File 2

Study invitation email sent 4/4/2023-8/31/2023

Dear [Veteran name],

My name is Alan Teo. I am a VA doctor and researcher, and **my mission** is to help Veterans be more connected with each other and with the VA.

I am working with a **team of Veterans and scientists** at [VA name] on an important new research project called **"Buddy Check Postcard Project"** that you may be interested in.

We are reaching out to Veterans like you who are over age 60 and have not been seen in primary care or mental health at the [VA name] recently.

We want to see how you and your peers are doing, and we want to see if sending you postcards can help you feel more connected. **Basically, it is like a Buddy Check.**

If you join this study, here's what will happen:

- We will send you some postcards in the mail.
- We will send you four short surveys (15 minutes each) over the course of one year.
- You will receive a $20 gift card for completing each survey, plus an extra $20 if you complete all the surveys for your time and participation. That's a total of $100.

Participating in this study is a great way to **help older Veterans who may be struggling with staying connected.**

Are you willing to learn more?

Yes. Take me to the screening questions to see if I am eligible.

Maybe. I want to read more about the study so I can decide if I'm interested before answering any questions.

No. I am definitely not interested. Do not contact me again.

If you have any questions, feel free to call [name] at [phone number] and leave a message with your contact information. Alternatively, if you would like to confirm that this is a valid VA study, you may contact the VA Central Institutional Review Board (IRB) toll free at [phone number].

Sincerely,

[Signature block]

Alan Teo, M.D., M.S.

Study invitation email sent 9/1/2023-1/2/2024

Buddy Check Postcard Project

Dear [Veteran name],

I am a VA doctor, researcher, and a university professor. I am working with a **team of Veterans and doctors at [insert VA name].**

Our mission is to help Veterans be more connected with each other and with the VA. I am writing you today about a VA research study opportunity that will help us achieve that mission.

You are one of a small number of Veterans we chose to invite to an important new project called "Buddy Check Postcard Project." We are inviting you because you were born before 1974 and have previously been seen at the [insert VA name]. We want to see how you and your peers are doing, and we want to see if occasional check-ins will be helpful. Basically, it is like a Buddy Check.

If you join this study, here's what will happen:

- We will send you some postcards in the mail.
- We will send you four short surveys (15 minutes each) over the course of one year.
- **You will receive up to $100** for completing the surveys: a $20 gift card for each survey, plus an extra $20 if you complete all four surveys.

Participating in this study is a great way to help other Veterans. By joining, you will contribute to new research and help us improve VA services.

Are you willing to help us by joining the Buddy Check Postcard Project?

Yes. Take me to the confidential screening questions to see if I am eligible.
Maybe. I want to read more about the study so I can decide if I'm interested before answering any questions.

No. I am definitely not interested. Do not contact me again.

Sincerely,

[signature block]

Dr. Alan Teo, M.D., M.S.
Director of Education, VA Center to Improve Veteran Involvement in Care
Associate Professor, Department of Psychiatry, Oregon Health & Science University

[Team photo]

This email is being securely sent to you by Qualtrics. You can confirm who we are by visiting our VA website: [link]

If you have any questions, please contact the Buddy Check Postcard Project team at [phone number] or email us at [@va.gov]. Please do not include your personal information or health information in email.

If you would like to confirm that this is a valid VA study, you may contact the VA Central Institutional Review Board (IRB) toll free at [phone number].

If we do not hear from you, we may reach out to make sure you received this.

Study invitation email sent 1/3/2024-11/29/2024

Buddy Check Postcard Project

Dear [Veteran name],

I am a VA doctor, researcher, and a university professor. I am working with a **team of Veterans and doctors at [VA name].**

Our mission is to provide meaningful connections to Veterans with the VA. I am writing you today about a VA research study opportunity that will help us achieve that mission.

You are one of a small number of Veterans we chose to invite to an important new project called "Buddy Check Postcard Project." We are inviting you because you were born before 1974 and have previously been seen at the [VA name].

If you join this study, here's what will happen:

- We will send you some postcards in the mail with messages from your local VA.
- We will send you four short surveys (15 minutes each) over the course of one year.
- **You will receive up to $100** for completing the surveys: a $20 gift card for each survey, plus an extra $20 if you complete all four surveys.

All you need to do in this study is to receive the postcards and fill out the surveys. We want to see if the postcards we send you are helpful. It is a chance for us to do a Buddy Check with you.

Participating in this study allows us to help other Veterans in the future. By joining, you will contribute to new research and help us improve VA services.

Are you willing to help us by joining the Buddy Check Postcard Project?

Yes. Take me to the confidential screening questions to see if I am eligible.

Maybe. I want to read more about the study so I can decide if I’m interested before answering any questions.

No. I am definitely not interested. Do not contact me again.

Sincerely,

[signature block]

Dr. Alan Teo, M.D., M.S.
Director of Education, VA Center to Improve Veteran Involvement in Care
Associate Professor, Department of Psychiatry, Oregon Health & Science University

[Team photo]

This email is being securely sent to you by Qualtrics. You can confirm who we are by visiting our VA website: [link]

If you have any questions, please contact the Buddy Check Postcard Project team at [phone number] or email us at [@va.gov]. Please do not include your personal information or health information in email.

If you would like to confirm that this is a valid VA study, you may contact the VA Central Institutional Review Board (IRB) toll free at [phone number].

If we do not hear from you, we may reach out to make sure you received this.
