# Supplementary material for: Enhancing reach and effectiveness of recruitment through email: a study within a trial (SWAT)
Source: Trials. 2026 Aug 6;27:643. doi: 10.1186/s13063-026-09902-1 (PMC13729685; doi:10.1186/s13063-026-09902-1)
Supplement: Supplementary file 2 [file 13063_2026_9902_MOESM3_ESM.pdf]

Additional File 3

Prenotification email

U.S. Department of Veterans Affairs

Buddy Check Postcard Project

Dear [Veteran name],

Soon you will receive an invitation to join an important new VA study called "Buddy Check Postcard Project."

The VA Buddy Check Postcard Project team will send you the invitation from the email address: [@qualtrics-research.com]. Qualtrics is an online survey platform, partnering with the VA to securely send your invitation by email.

I am leading this project with a **team of Veterans and VA doctors**. This research study is being supported by the VA to improve services for Veterans like you. You will receive up to **$100** for your time and participation.

To find out more about who we are, you can visit our website: [link]

Or feel free to send us an email at: [@va.gov]

Sincerely,

[signature block]

Dr. Alan Teo, M.D., M.S.
Director of Education, VA Center to Improve Veteran Involvement in Care
Associate Professor, Department of Psychiatry, Oregon Health & Science University

[Team Photo]
