# Supplementary material for: Enhancing reach and effectiveness of recruitment through email: a study within a trial (SWAT)
Source: Trials. 2026 Aug 6;27:643. doi: 10.1186/s13063-026-09902-1 (PMC13729685; doi:10.1186/s13063-026-09902-1)
Supplement: Supplementary file 3 [file 13063_2026_9902_MOESM4_ESM.pdf]

Supplemental Sensitivity Analyses

1. Alternative definition of "effectiveness": baseline survey completion within 5 days

- the first model looks at age continuously and uses institutional as the subject line reference category for odds ratios

```
. logit outc_blcompletion_5d i.subject_line i.prenot_email i.site Age_EmailDate i.sex i.rurality_2c
> at i.ethnic_race if SampleFix==1, or

Iteration 0:  Log likelihood = -336.75347
Iteration 1:  Log likelihood = -317.36568
Iteration 2:  Log likelihood = -306.06746
Iteration 3:  Log likelihood = -305.89738
Iteration 4:  Log likelihood = -305.89721
Iteration 5:  Log likelihood = -305.89721

Logistic regression                                     Number of obs =  1,945
                                                        LR chi2(10)   =  61.71
                                                        Prob > chi2   = 0.0000
Log likelihood = -305.89721                             Pseudo R2     = 0.0916

--------------------------------------------------------------------------------------
  outc_blcompletion_5d | Odds ratio   Std. err.      z    P>|z|     [95% conf. interval]
-----------------------+--------------------------------------------------------------
          subject_line |
             Altruism |     .41531   .1973495    -1.85   0.064     .1636415    1.054026
            Incentive |   .6663746   .2595882    -1.04   0.297     .3105492    1.429903
                       |
        1.prenot_email |   1.847995   .5205084     2.18   0.029     1.064021    3.209602
                       |
                  site |
                  PDX |   1.139802    .335909     0.44   0.657      .639696    2.030886
                  SEA |   .8761729   .2571745    -0.45   0.652     .4928843    1.557524
                       |
         Age_EmailDate |   .9620949   .0121193    -3.07   0.002     .9386323     .986144
                       |
                   sex |
               Female |   2.937398   .8277353     3.82   0.000     1.690834    5.102988
                       |
         rurality_2cat |
                Rural |   1.107322   .2652273     0.43   0.670     .6924573     1.77074
                       |
           ethnic_race |
Non-White or Hispanic |   .2770066   .1323532    -2.69   0.007     .1085895    .7066307
              Unknown |   .4981363   .2042888    -1.70   0.089     .2229796    1.112836
                       |
                 _cons |   .5293118   .4550399    -0.74   0.459     .0981632    2.854134
--------------------------------------------------------------------------------------
Note: _cons estimates baseline odds.
```

- the second model looks at age continuously and uses altruism as the subject line reference category for odds ratios

```
. logit outc_blcompletion_5d ib2.subject_line i.prenot_email i.site Age_EmailDate i.sex i.rurality_
> 2cat i.ethnic_race if SampleFix==1, or

Iteration 0:  Log likelihood = -336.75347
Iteration 1:  Log likelihood = -317.36568
Iteration 2:  Log likelihood = -306.06746
Iteration 3:  Log likelihood = -305.89738
```

```
Iteration 4:  Log likelihood = -305.89721
Iteration 5:  Log likelihood = -305.89721

Logistic regression                                     Number of obs =  1,945
                                                        LR chi2(10)   =  61.71
                                                        Prob > chi2   = 0.0000
Log likelihood = -305.89721                             Pseudo R2     = 0.0916

-------------------------------------------------------------------------------------
  outc_blcompletion_5d | Odds ratio   Std. err.      z    P>|z|     [95% conf. interval]
----------------------+--------------------------------------------------------------
          subject_line |
         Institutional |   2.40784   1.144172     1.85   0.064     .9487428    6.11092
             Incentive |  1.604523    .833841     0.91   0.363     .5794184    4.443239
                       |
        1.prenot_email |  1.847995   .5205084     2.18   0.029     1.064021    3.209602
                       |
                  site |
                   PDX |  1.139802    .335909     0.44   0.657      .639696    2.030886
                   SEA |  .8761729   .2571745    -0.45   0.652     .4928843    1.557524
                       |
         Age_EmailDate |  .9620949   .0121193    -3.07   0.002     .9386323     .986144
                       |
                   sex |
                Female |  2.937398   .8277353     3.82   0.000     1.690834    5.102988
                       |
         rurality_2cat |
                 Rural |  1.107322   .2652273     0.43   0.670     .6924573     1.77074
                       |
           ethnic_race |
Non-White or Hispanic |  .2770066   .1323532    -2.69   0.007     .1085895    .7066307
               Unknown |  .4981363   .2042888    -1.70   0.089     .2229796    1.112836
                       |
                 _cons |  .2198285   .2029958    -1.64   0.101       .03598    1.343097
-------------------------------------------------------------------------------------
Note: _cons estimates baseline odds.
```

- the third model looks at age continuously and uses incentive as the subject line reference cateogry for odds ratios

```
. logit outc_blcompletion_5d ib3.subject_line i.prenot_email i.site Age_EmailDate i.sex i.rurality_
> 2cat i.ethnic_race if SampleFix==1, or

Iteration 0:  Log likelihood = -336.75347
Iteration 1:  Log likelihood = -317.36568
Iteration 2:  Log likelihood = -306.06746
Iteration 3:  Log likelihood = -305.89738
Iteration 4:  Log likelihood = -305.89721
Iteration 5:  Log likelihood = -305.89721

Logistic regression                                     Number of obs =  1,945
                                                        LR chi2(10)   =  61.71
                                                        Prob > chi2   = 0.0000
Log likelihood = -305.89721                             Pseudo R2     = 0.0916

-------------------------------------------------------------------------------------
  outc_blcompletion_5d | Odds ratio   Std. err.      z    P>|z|     [95% conf. interval]
----------------------+--------------------------------------------------------------
          subject_line |
         Institutional |  1.500657   .5845855     1.04   0.297     .6993483    3.220102
             Altruism |  .6232381   .3238853    -0.91   0.363      .225061    1.725868
                       |
        1.prenot_email |  1.847995   .5205084     2.18   0.029     1.064021    3.209602
                       |
                  site |
```

```
                 PDX |   1.139802    .335909     0.44   0.657     .639696    2.030886
                 SEA |   .8761729   .2571745    -0.45   0.652    .4928843    1.557524
                     |
       Age_EmailDate |   .9620949   .0121193    -3.07   0.002    .9386323     .986144
                     |
                 sex |
              Female |   2.937398   .8277353     3.82   0.000    1.690834    5.102988
                     |
       rurality_2cat |
               Rural |   1.107322   .2652273     0.43   0.670    .6924573     1.77074
                     |
         ethnic_race |
Non-White or Hispanic |   .2770066   .1323532    -2.69   0.007    .1085895    .7066307
             Unknown |   .4981363   .2042888    -1.70   0.089    .2229796    1.112836
                     |
               _cons |     .35272   .3304063    -1.11   0.266    .0562441     2.21199
-----------------------------------------------------------------------------------------
Note: _cons estimates baseline odds.
```

- the fourth model looks at age categorically and uses institutional as the subject line reference category for predicted probabilities

```
. logit outc_blcompletion_5d i.subject_line i.prenot_email i.site i.age_cat2 i.sex i.rurality_2cat
> i.ethnic_race if SampleFix==1, or

Iteration 0:  Log likelihood = -336.75347
Iteration 1:  Log likelihood =   -317.339
Iteration 2:  Log likelihood = -305.38343
Iteration 3:  Log likelihood = -305.20382
Iteration 4:  Log likelihood = -305.20363
Iteration 5:  Log likelihood = -305.20363

Logistic regression                                     Number of obs =  1,945
                                                        LR chi2(12)   =  63.10
                                                        Prob > chi2   = 0.0000
Log likelihood = -305.20363                             Pseudo R2     = 0.0937

-----------------------------------------------------------------------------------------
  outc_blcompletion_5d | Odds ratio   Std. err.      z    P>|z|     [95% conf. interval]
-----------------------+-----------------------------------------------------------------
          subject_line |
              Altruism |   .4192507   .1992644    -1.83   0.067    .1651615    1.064238
             Incentive |   .6678973   .2622644    -1.03   0.304    .3093619    1.441958
                       |
        1.prenot_email |   1.850333   .5213342     2.18   0.029    1.065179    3.214233
                       |
                  site |
                   PDX |   1.159245   .3425765     0.50   0.617    .6495772    2.068805
                   SEA |   .8894367    .261785    -0.40   0.691    .4995554    1.583603
                       |
              age_cat2 |
               Sixties |   .7272405   .2049996    -1.13   0.259    .4185385    1.263632
             Seventies |   .4170421   .1332348    -2.74   0.006    .2229677     .780042
         Eighties plus |   .2841772   .1556939    -2.30   0.022    .0971038     .831653
                       |
                   sex |
                Female |   2.860091   .8138999     3.69   0.000     1.63739    4.995827
                       |
         rurality_2cat |
                 Rural |   1.120915   .2686639     0.48   0.634    .7007356    1.793044
                       |
           ethnic_race |
 Non-White or Hispanic |   .2800347   .1337439    -2.67   0.008    .1098197    .7140748
               Unknown |   .4988375   .2046415    -1.70   0.090    .2232363    1.114688
```

```
                                |
                  _cons |    .063977   .0217398    -8.09   0.000     .0328686    .1245281
------------------------------------------------------------------------------------------
Note: _cons estimates baseline odds.

. margins subject_line

Predictive margins                                          Number of obs = 1,945
Model VCE: OIM

Expression: Pr(outc_blcompletion_5d), predict()

--------------------------------------------------------------------------------
               |            Delta-method
               |     Margin   std. err.      z    P>|z|     [95% conf. interval]
---------------+----------------------------------------------------------------
  subject_line |
 Institutional |   .0483184   .0064916     7.44   0.000      .035595    .0610418
      Altruism |   .0213154   .0089156     2.39   0.017     .0038411    .0387897
     Incentive |   .0332015   .0106209     3.13   0.002     .0123849     .054018
--------------------------------------------------------------------------------

. margins prenot_email

Predictive margins                                          Number of obs = 1,945
Model VCE: OIM

Expression: Pr(outc_blcompletion_5d), predict()

------------------------------------------------------------------------------
             |            Delta-method
             |     Margin   std. err.      z    P>|z|     [95% conf. interval]
-------------+----------------------------------------------------------------
prenot_email |
           0 |   .0319119    .005431     5.88   0.000     .0212674    .0425564
           1 |   .0564131   .0095929     5.88   0.000     .0376113    .0752148
------------------------------------------------------------------------------

. margins age_cat2

Predictive margins                                          Number of obs = 1,945
Model VCE: OIM

Expression: Pr(outc_blcompletion_5d), predict()

--------------------------------------------------------------------------------
               |            Delta-method
               |     Margin   std. err.      z    P>|z|     [95% conf. interval]
---------------+----------------------------------------------------------------
      age_cat2 |
       Fifties |   .0629714   .0112653     5.59   0.000     .0408918     .085051
       Sixties |   .0470917   .0087195     5.40   0.000     .0300019    .0641816
     Seventies |    .027931   .0065024     4.30   0.000     .0151865    .0406754
 Eighties plus |   .0193235   .0095161     2.03   0.042     .0006722    .0379748
--------------------------------------------------------------------------------

. margins sex

Predictive margins                                          Number of obs = 1,945
Model VCE: OIM

Expression: Pr(outc_blcompletion_5d), predict()

------------------------------------------------------------------------------
             |            Delta-method
             |     Margin   std. err.      z    P>|z|     [95% conf. interval]
```

```
-------------+----------------------------------------------------------------
         sex |
       Male  |   .0353882   .0044023     8.04   0.000     .0267598    .0440166
     Female  |   .0916706   .0198502     4.62   0.000      .052765    .1305762
------------------------------------------------------------------------------

. margins rurality_2cat

Predictive margins                                       Number of obs = 1,945
Model VCE: OIM

Expression: Pr(outc_blcompletion_5d), predict()

-------------------------------------------------------------------------------
              |            Delta-method
              |     Margin   std. err.      z    P>|z|     [95% conf. interval]
--------------+----------------------------------------------------------------
rurality_2cat |
        Urban |   .0400128   .0054998     7.28   0.000     .0292335    .0507921
        Rural |   .0444278   .0075562     5.88   0.000      .029618    .0592377
-------------------------------------------------------------------------------

. margins ethnic_race

Predictive margins                                       Number of obs = 1,945
Model VCE: OIM

Expression: Pr(outc_blcompletion_5d), predict()

----------------------------------------------------------------------------------------
                       |            Delta-method
                       |     Margin   std. err.      z    P>|z|     [95% conf. interval]
-----------------------+----------------------------------------------------------------
           ethnic_race |
    Non-Hispanic White |   .0509089   .0058843     8.65   0.000      .039376    .0624419
Non-White or Hispanic  |   .0152872    .006802     2.25   0.025     .0019555    .0286189
               Unknown |   .0266289   .0098614     2.70   0.007     .0073008     .045957
----------------------------------------------------------------------------------------
```

---

2. Impact of change in sender email domain

Variable domain_change was defined such that 0 was the first email domain (@qemailserver.com), and 1 was the second email domain (@qualtrics-research.com).

Primary outcome: reach

- the first model looks at age continuously and uses institutional as the subject line reference category for odds ratios

```
. logit outc_open_email i.domain_change i.subject_line i.prenot_email i.site Age_EmailDate i.sex i.
> rurality_2cat i.ethnic_race if SampleFix==1, or

Iteration 0:  Log likelihood = -1348.0353
Iteration 1:  Log likelihood =  -1315.045
Iteration 2:  Log likelihood = -1315.0038
Iteration 3:  Log likelihood = -1315.0038

Logistic regression                                     Number of obs =  1,945
                                                        LR chi2(11)   =  66.06
                                                        Prob > chi2   = 0.0000
Log likelihood = -1315.0038                             Pseudo R2     = 0.0245
```

```
------------------------------------------------------------------------------------------
       outc_open_email | Odds ratio   Std. err.      z    P>|z|     [95% conf. interval]
-----------------------+------------------------------------------------------------------
         domain_change |
   second email domain |   .4749618   .1230067    -2.87   0.004     .2858989    .7890504
                       |
          subject_line |
             Altruism  |   .4340231   .0681528    -5.32   0.000     .3190453    .5904367
            Incentive  |   .3088727   .0899099    -4.04   0.000     .1745833    .5464572
                       |
        1.prenot_email |   .9883036   .1209439    -0.10   0.923     .7775429    1.256193
                       |
                  site |
                  PDX  |   1.609348   .2032549     3.77   0.000     1.256453    2.061359
                  SEA  |   1.582036   .1883515     3.85   0.000     1.252781    1.997824
                       |
         Age_EmailDate |   1.000208   .0048391     0.04   0.966     .9907686    1.009738
                       |
                   sex |
               Female  |   .9806533   .1612082    -0.12   0.905     .7105386    1.353453
                       |
         rurality_2cat |
                Rural  |   .8144965   .0804013    -2.08   0.038     .6712197    .9883568
                       |
           ethnic_race |
Non-White or Hispanic  |   .6565627   .0864891    -3.19   0.001     .5071627    .8499729
              Unknown  |   .9934062   .1455734    -0.05   0.964     .7454046     1.32392
                       |
                 _cons |   2.065711   .9055482     1.65   0.098     .8748377    4.877663
------------------------------------------------------------------------------------------
Note: _cons estimates baseline odds.
```

- the second model looks at age continuously and uses altruism as the subject line reference category for odds ratios

```
. logit outc_open_email i.domain_change ib2.subject_line i.prenot_email i.site Age_EmailDate i.sex
> i.rurality_2cat i.ethnic_race if SampleFix==1, or

Iteration 0:  Log likelihood = -1348.0353
Iteration 1:  Log likelihood =  -1315.045
Iteration 2:  Log likelihood = -1315.0038
Iteration 3:  Log likelihood = -1315.0038

Logistic regression                                     Number of obs =  1,945
                                                        LR chi2(11)   =  66.06
                                                        Prob > chi2   = 0.0000
Log likelihood = -1315.0038                             Pseudo R2     = 0.0245

------------------------------------------------------------------------------------------
       outc_open_email | Odds ratio   Std. err.      z    P>|z|     [95% conf. interval]
-----------------------+------------------------------------------------------------------
         domain_change |
   second email domain |   .4749618   .1230067    -2.87   0.004     .2858989    .7890504
                       |
          subject_line |
        Institutional  |   2.304025    .361791     5.32   0.000     1.693662    3.134351
            Incentive  |   .7116502   .1742116    -1.39   0.165     .4404463    1.149848
                       |
        1.prenot_email |   .9883036   .1209439    -0.10   0.923     .7775429    1.256193
                       |
                  site |
                  PDX  |   1.609348   .2032549     3.77   0.000     1.256453    2.061359
                  SEA  |   1.582036   .1883515     3.85   0.000     1.252781    1.997824
                       |
```

```
         Age_EmailDate |   1.000208   .0048391     0.04   0.966     .9907686    1.009738
                       |
                   sex |
                Female |   .9806533   .1612082    -0.12   0.905     .7105386    1.353453
                       |
         rurality_2cat |
                 Rural |   .8144965   .0804013    -2.08   0.038     .6712197    .9883568
                       |
           ethnic_race |
  Non-White or Hispanic |   .6565627   .0864891    -3.19   0.001     .5071627    .8499729
               Unknown |   .9934062   .1455734    -0.05   0.964     .7454046     1.32392
                       |
                 _cons |   .8965665   .3643792    -0.27   0.788      .404241    1.988496
------------------------------------------------------------------------------------------
Note: _cons estimates baseline odds.
```

- the third model looks at age continuously and uses incentive as the subject line reference cateogry for odds ratios

```
. logit outc_open_email i.domain_change ib3.subject_line i.prenot_email i.site Age_EmailDate i.sex
> i.rurality_2cat i.ethnic_race if SampleFix==1, or

Iteration 0:  Log likelihood = -1348.0353
Iteration 1:  Log likelihood =  -1315.045
Iteration 2:  Log likelihood = -1315.0038
Iteration 3:  Log likelihood = -1315.0038

Logistic regression                                      Number of obs =  1,945
                                                         LR chi2(11)   =  66.06
                                                         Prob > chi2   = 0.0000
Log likelihood = -1315.0038                              Pseudo R2     = 0.0245

------------------------------------------------------------------------------------------
       outc_open_email | Odds ratio   Std. err.      z    P>|z|     [95% conf. interval]
-----------------------+------------------------------------------------------------------
         domain_change |
   second email domain |   .4749618   .1230067    -2.87   0.004     .2858989    .7890504
                       |
          subject_line |
         Institutional |    3.23758   .9424288     4.04   0.000      1.82997    5.727924
              Altruism |   1.405185   .3439884     1.39   0.165     .8696805    2.270425
                       |
        1.prenot_email |   .9883036   .1209439    -0.10   0.923     .7775429    1.256193
                       |
                  site |
                   PDX |   1.609348   .2032549     3.77   0.000     1.256453    2.061359
                   SEA |   1.582036   .1883515     3.85   0.000     1.252781    1.997824
                       |
         Age_EmailDate |   1.000208   .0048391     0.04   0.966     .9907686    1.009738
                       |
                   sex |
                Female |   .9806533   .1612082    -0.12   0.905     .7105386    1.353453
                       |
         rurality_2cat |
                 Rural |   .8144965   .0804013    -2.08   0.038     .6712197    .9883568
                       |
           ethnic_race |
  Non-White or Hispanic |   .6565627   .0864891    -3.19   0.001     .5071627    .8499729
               Unknown |   .9934062   .1455734    -0.05   0.964     .7454046     1.32392
                       |
                 _cons |   .6380418   .2348718    -1.22   0.222     .3101061    1.312768
------------------------------------------------------------------------------------------
Note: _cons estimates baseline odds.
```

- the fourth model looks at age categorically and uses institutional as the subject line reference category for predicted probabilities

```
. logit outc_open_email i.domain_change i.subject_line i.prenot_email i.site i.age_cat2 i.sex i.rur
> ality_2cat i.ethnic_race if SampleFix==1, or

Iteration 0:  Log likelihood = -1348.0353
Iteration 1:  Log likelihood = -1313.3142
Iteration 2:  Log likelihood = -1313.2657
Iteration 3:  Log likelihood = -1313.2657

Logistic regression                                     Number of obs =  1,945
                                                       LR chi2(13)   =  69.54
                                                       Prob > chi2   = 0.0000
Log likelihood = -1313.2657                             Pseudo R2     = 0.0258

--------------------------------------------------------------------------------------
      outc_open_email | Odds ratio   Std. err.      z    P>|z|     [95% conf. interval]
----------------------+---------------------------------------------------------------
        domain_change |
  second email domain |   .4766651   .1234377    -2.86   0.004     .2869361    .7918474
                      |
         subject_line |
            Altruism  |   .4353116   .0684236    -5.29   0.000     .3198937    .5923723
           Incentive  |   .3193226   .0931393    -3.91   0.000     .1802823    .5655958
                      |
       1.prenot_email |    .984451   .1205887    -0.13   0.898     .7743327    1.251586
                      |
                 site |
                 PDX  |   1.633248    .207169     3.87   0.000     1.273743    2.094222
                 SEA  |   1.603318   .1916106     3.95   0.000     1.268509    2.026496
                      |
             age_cat2 |
             Sixties  |   .7876389   .1030165    -1.83   0.068     .6095329    1.017788
           Seventies  |   .8940473   .1160891    -0.86   0.388      .693162    1.153151
       Eighties plus  |   .9098135   .1592841    -0.54   0.589     .6455497    1.282257
                      |
                  sex |
              Female  |   .9950514   .1645336    -0.03   0.976      .719611     1.37592
                      |
        rurality_2cat |
               Rural  |    .809784    .080076    -2.13   0.033     .6671103    .9829712
                      |
          ethnic_race |
Non-White or Hispanic  |    .6632763   .0874835    -3.11   0.002     .5121821    .8589435
             Unknown  |   .9982175   .1464287    -0.01   0.990     .7487939    1.330724
                      |
                _cons |     2.3211   .6999076     2.79   0.005      1.28535     4.19147
--------------------------------------------------------------------------------------
Note: _cons estimates baseline odds.

. margins subject_line

Predictive margins                                       Number of obs = 1,945
Model VCE: OIM

Expression: Pr(outc_open_email), predict()

--------------------------------------------------------------------------------
              |            Delta-method
              |     Margin   std. err.      z    P>|z|     [95% conf. interval]
--------------+-----------------------------------------------------------------
 subject_line |
Institutional  |   .5940654   .0211499    28.09   0.000     .5526124    .6355185
    Altruism  |   .3983359   .0270875    14.71   0.000     .3452454    .4514264
```

```
   Incentive |   .3299502    .042677     7.73   0.000     .2463048    .4135956
------------------------------------------------------------------------------

. margins prenot_email

Predictive margins                                       Number of obs = 1,945
Model VCE: OIM

Expression: Pr(outc_open_email), predict()

----------------------------------------------------------------------------
             |            Delta-method
             |     Margin   std. err.      z    P>|z|     [95% conf. interval]
-------------+--------------------------------------------------------------
prenot_email |
          0  |   .4953053   .0146467    33.82   0.000     .4665983    .5240122
          1  |   .4915272   .0228876    21.48   0.000     .4466683    .5363861
----------------------------------------------------------------------------

. margins age_cat2

Predictive margins                                       Number of obs = 1,945
Model VCE: OIM

Expression: Pr(outc_open_email), predict()

------------------------------------------------------------------------------
               |            Delta-method
               |     Margin   std. err.      z    P>|z|     [95% conf. interval]
---------------+--------------------------------------------------------------
     age_cat2 |
      Fifties |   .5241317    .024218    21.64   0.000     .4766653     .571598
      Sixties |   .4664935   .0202063    23.09   0.000     .4268899    .5060971
    Seventies |   .4970826   .0189587    26.22   0.000     .4599242    .5342411
Eighties plus  |   .5013081   .0343039    14.61   0.000     .4340737    .5685424
------------------------------------------------------------------------------

. margins sex

Predictive margins                                       Number of obs = 1,945
Model VCE: OIM

Expression: Pr(outc_open_email), predict()

----------------------------------------------------------------------------
             |            Delta-method
             |     Margin   std. err.      z    P>|z|     [95% conf. interval]
-------------+--------------------------------------------------------------
         sex |
       Male  |   .4941942   .0116912    42.27   0.000     .4712799    .5171086
     Female  |   .4929978   .0379866    12.98   0.000     .4185453    .5674502
----------------------------------------------------------------------------

. margins rurality_2cat

Predictive margins                                       Number of obs = 1,945
Model VCE: OIM

Expression: Pr(outc_open_email), predict()

-----------------------------------------------------------------------------
              |            Delta-method
              |     Margin   std. err.      z    P>|z|     [95% conf. interval]
--------------+--------------------------------------------------------------
rurality_2cat |
       Urban  |   .5117369   .0138521    36.94   0.000     .4845874    .5388865
```

```
      Rural  |   .4609073   .0190256    24.23   0.000     .4236178    .4981967
------------------------------------------------------------------------------

. margins ethnic_race

Predictive margins                                       Number of obs = 1,945
Model VCE: OIM

Expression: Pr(outc_open_email), predict()

-----------------------------------------------------------------------------------------
                      |            Delta-method
                      |     Margin   std. err.      z    P>|z|     [95% conf. interval]
----------------------+------------------------------------------------------------------
          ethnic_race |
   Non-Hispanic White |   .5104574   .0132625    38.49   0.000     .4844634    .5364515
Non-White or Hispanic |   .4118395   .0278276    14.80   0.000     .3572985    .4663806
              Unknown |   .5100254   .0329117    15.50   0.000     .4455197    .5745311
-----------------------------------------------------------------------------------------
```

Secondary outcome: effectiveness

- the first model looks at age continuously and uses institutional as the subject line reference category for odds ratios

```
. logit outc_blcompletion_10d i.domain_change i.subject_line i.prenot_email i.site Age_EmailDate i.
> sex i.rurality_2cat i.ethnic_race if SampleFix==1, or

Iteration 0:  Log likelihood = -388.31744
Iteration 1:  Log likelihood = -364.20049
Iteration 2:  Log likelihood = -354.33198
Iteration 3:  Log likelihood = -354.23174
Iteration 4:  Log likelihood = -354.23159
Iteration 5:  Log likelihood = -354.23159

Logistic regression                                     Number of obs =  1,945
                                                        LR chi2(11)   =  68.17
                                                        Prob > chi2   = 0.0000
Log likelihood = -354.23159                             Pseudo R2     = 0.0878

-----------------------------------------------------------------------------------------
 outc_blcompletion_10d | Odds ratio   Std. err.      z    P>|z|     [95% conf. interval]
-----------------------+-----------------------------------------------------------------
         domain_change |
   second email domain |   .5139325   .3854013    -0.89   0.375      .118191    2.234743
                       |
          subject_line |
             Altruism  |   .3353924   .1674375    -2.19   0.029     .1260692    .8922724
            Incentive  |   .3619337   .2983105    -1.23   0.218     .0719544    1.820541
                       |
        1.prenot_email |   1.562486   .3996306     1.74   0.081     .9464721    2.579434
                       |
                  site |
                   PDX |   1.291726   .3589611     0.92   0.357     .7492517    2.226963
                   SEA |    1.08201   .2942282     0.29   0.772     .6349891    1.843726
                       |
         Age_EmailDate |   .9534269      .0112    -4.06   0.000      .931726    .9756331
                       |
                   sex |
                Female |   2.692801   .7074697     3.77   0.000     1.609057    4.506479
                       |
         rurality_2cat |
                 Rural |    1.18619    .258768     0.78   0.434     .7735066    1.819049
                       |
```

```
           ethnic_race |
Non-White or Hispanic  |   .3208068   .1314611    -2.77   0.006     .1436931    .7162279
               Unknown |   .5382313   .1970428    -1.69   0.091     .2626333    1.103032
                       |
                 _cons |   2.115205   2.311619     0.69   0.493     .2483761    18.01337
-----------------------------------------------------------------------------------------
Note: _cons estimates baseline odds.
```

- the second model looks at age continuously and uses altruism as the subject line reference category for odds ratios

```
. logit outc_blcompletion_10d i.domain_change ib2.subject_line i.prenot_email i.site Age_EmailDate
> i.sex i.rurality_2cat i.ethnic_race if SampleFix==1, or

Iteration 0:  Log likelihood = -388.31744
Iteration 1:  Log likelihood = -364.20049
Iteration 2:  Log likelihood = -354.33198
Iteration 3:  Log likelihood = -354.23174
Iteration 4:  Log likelihood = -354.23159
Iteration 5:  Log likelihood = -354.23159

Logistic regression                                     Number of obs =  1,945
                                                        LR chi2(11)   =  68.17
                                                        Prob > chi2   = 0.0000
Log likelihood = -354.23159                             Pseudo R2     = 0.0878

-----------------------------------------------------------------------------------------
 outc_blcompletion_10d | Odds ratio   Std. err.      z    P>|z|     [95% conf. interval]
-----------------------+-----------------------------------------------------------------
         domain_change |
   second email domain |   .5139325   .3854013    -0.89   0.375      .118191    2.234743
                       |
          subject_line |
         Institutional |   2.981582   1.488491     2.19   0.029     1.120734    7.932151
             Incentive |   1.079135   .7078259     0.12   0.908     .2983705    3.902974
                       |
        1.prenot_email |   1.562486   .3996306     1.74   0.081     .9464721    2.579434
                       |
                  site |
                   PDX |   1.291726   .3589611     0.92   0.357     .7492517    2.226963
                   SEA |    1.08201   .2942282     0.29   0.772     .6349891    1.843726
                       |
         Age_EmailDate |   .9534269      .0112    -4.06   0.000      .931726    .9756331
                       |
                   sex |
                Female |   2.692801   .7074697     3.77   0.000     1.609057    4.506479
                       |
         rurality_2cat |
                 Rural |    1.18619    .258768     0.78   0.434     .7735066    1.819049
                       |
           ethnic_race |
Non-White or Hispanic  |   .3208068   .1314611    -2.77   0.006     .1436931    .7162279
               Unknown |   .5382313   .1970428    -1.69   0.091     .2626333    1.103032
                       |
                 _cons |   .7094237   .6862799    -0.35   0.723     .1065303    4.724307
-----------------------------------------------------------------------------------------
Note: _cons estimates baseline odds.
```

- the third model looks at age continuously and uses incentive as the subject line reference cateogry for odds ratios

```
. logit outc_blcompletion_10d i.domain_change ib3.subject_line i.prenot_email i.site Age_EmailDate
> i.sex i.rurality_2cat i.ethnic_race if SampleFix==1, or
```

```
Iteration 0:  Log likelihood = -388.31744
Iteration 1:  Log likelihood = -364.20049
Iteration 2:  Log likelihood = -354.33198
Iteration 3:  Log likelihood = -354.23174
Iteration 4:  Log likelihood = -354.23159
Iteration 5:  Log likelihood = -354.23159

Logistic regression                                     Number of obs =  1,945
                                                        LR chi2(11)   =  68.17
                                                        Prob > chi2   = 0.0000
Log likelihood = -354.23159                             Pseudo R2     = 0.0878

-----------------------------------------------------------------------------------------
 outc_blcompletion_10d | Odds ratio   Std. err.      z    P>|z|     [95% conf. interval]
----------------------+------------------------------------------------------------------
         domain_change |
   second email domain |    .5139325   .3854013    -0.89   0.375      .118191    2.234743
                       |
          subject_line |
         Institutional |    2.762937   2.277249     1.23   0.218     .5492873    13.89769
              Altruism |    .9266682   .6078199    -0.12   0.908     .2562149    3.351538
                       |
        1.prenot_email |    1.562486   .3996306     1.74   0.081     .9464721    2.579434
                       |
                  site |
                   PDX |    1.291726   .3589611     0.92   0.357     .7492517    2.226963
                   SEA |     1.08201   .2942282     0.29   0.772     .6349891    1.843726
                       |
          Age_EmailDate |    .9534269      .0112    -4.06   0.000      .931726    .9756331
                       |
                   sex |
                Female |    2.692801   .7074697     3.77   0.000     1.609057    4.506479
                       |
         rurality_2cat |
                 Rural |     1.18619    .258768     0.78   0.434     .7735066    1.819049
                       |
           ethnic_race |
Non-White or Hispanic |    .3208068   .1314611    -2.77   0.006     .1436931    .7162279
               Unknown |    .5382313   .1970428    -1.69   0.091     .2626333    1.103032
                       |
                 _cons |    .7655639   .6598233    -0.31   0.757     .1413673    4.145854
-----------------------------------------------------------------------------------------
Note: _cons estimates baseline odds.
```

- the fourth model looks at age categorically and uses institutional as the subject line reference category for predicted probabilities

```
. logit outc_blcompletion_10d i.domain_change i.subject_line i.prenot_email i.site i.age_cat2 i.sex
>  i.rurality_2cat i.ethnic_race if SampleFix==1, or

Iteration 0:  Log likelihood = -388.31744
Iteration 1:  Log likelihood =  -364.0819
Iteration 2:  Log likelihood = -353.30419
Iteration 3:  Log likelihood = -353.20118
Iteration 4:  Log likelihood =   -353.201
Iteration 5:  Log likelihood =   -353.201

Logistic regression                                     Number of obs =  1,945
                                                        LR chi2(13)   =  70.23
                                                        Prob > chi2   = 0.0000
Log likelihood = -353.201                               Pseudo R2     = 0.0904

-----------------------------------------------------------------------------------------
 outc_blcompletion_10d | Odds ratio   Std. err.      z    P>|z|     [95% conf. interval]
```

domain_change						
second email domain	.5157649	.386589	-0.88	0.377	.1186965	2.241123
subject_line						
Altruism	.3392742	.1694136	-2.16	0.030	.1275002	.9027984
Incentive	.366243	.3022466	-1.22	0.224	.0726615	1.84601
1.prenot_email	1.564135	.4002201	1.75	0.080	.9472714	2.582699
site						
PDX	1.317034	.3669641	0.99	0.323	.762829	2.273875
SEA	1.101551	.3003722	0.35	0.723	.6455028	1.879799
age_cat2						
Sixties	.6793956	.1728588	-1.52	0.129	.412621	1.11865
Seventies	.3183676	.0960676	-3.79	0.000	.1762294	.5751476
Eighties plus	.2622813	.1290145	-2.72	0.007	.1000153	.6878092
sex						
Female	2.601046	.689411	3.61	0.000	1.547162	4.372807
rurality_2cat						
Rural	1.205572	.2632254	0.86	0.392	.7858529	1.84946
ethnic_race						
Non-White or Hispanic	.3256697	.133397	-2.74	0.006	.1459211	.7268363
Unknown	.5408024	.1979965	-1.68	0.093	.263876	1.108351
_cons	.155035	.1259792	-2.29	0.022	.0315323	.7622615

Note: _cons estimates baseline odds.

. margins subject_line

Predictive margins Number of obs = 1,945
Model VCE: OIM

Expression: Pr(outc_blcompletion_10d), predict()

	Margin	Delta-method std. err.	z	P>|z|	[95% conf.	interval]
subject_line						
Institutional	.0692824	.0178877	3.87	0.000	.0342231	.1043417
Altruism	.0254053	.0090937	2.79	0.005	.007582	.0432285
Incentive	.0273312	.0149031	1.83	0.067	-.0018784	.0565408

. margins prenot_email

Predictive margins Number of obs = 1,945
Model VCE: OIM

Expression: Pr(outc_blcompletion_10d), predict()

	Margin	Delta-method std. err.	z	P>|z|	[95% conf.	interval]
prenot_email						
0	.0420496	.0061692	6.82	0.000	.0299583	.054141
1	.063224	.0099695	6.34	0.000	.0436841	.0827638

. margins age_cat2

Predictive margins Number of obs = 1,945
Model VCE: OIM

Expression: Pr(outc_blcompletion_10d), predict()

	Margin	Delta-method std. err.	z	P>|z|	[95% conf.	interval]
age_cat2						
Fifties	.082908	.0131604	6.30	0.000	.0571141	.108702
Sixties	.0585585	.0096579	6.06	0.000	.0396293	.0774878
Seventies	.0287643	.0065214	4.41	0.000	.0159825	.041546
Eighties plus	.0238798	.0105062	2.27	0.023	.003288	.0444716

. margins sex

Predictive margins Number of obs = 1,945
Model VCE: OIM

Expression: Pr(outc_blcompletion_10d), predict()

	Margin	Delta-method std. err.	z	P>|z|	[95% conf.	interval]
sex						
Male	.0436709	.0048688	8.97	0.000	.0341282	.0532136
Female	.1025658	.0205449	4.99	0.000	.0622986	.142833

. margins rurality_2cat

Predictive margins Number of obs = 1,945
Model VCE: OIM

Expression: Pr(outc_blcompletion_10d), predict()

	Margin	Delta-method std. err.	z	P>|z|	[95% conf.	interval]
rurality_2cat						
Urban	.0471871	.0059364	7.95	0.000	.035552	.0588222
Rural	.0558942	.0084431	6.62	0.000	.039346	.0724425

. margins ethnic_race

Predictive margins Number of obs = 1,945
Model VCE: OIM

Expression: Pr(outc_blcompletion_10d), predict()

	Margin	Delta-method std. err.	z	P>|z|	[95% conf.	interval]
ethnic_race						
Non-Hispanic White	.0605445	.0063763	9.50	0.000	.0480473	.0730418
Non-White or Hispanic	.0212358	.0079688	2.66	0.008	.0056173	.0368543
Unknown	.0343959	.0111911	3.07	0.002	.0124618	.05633
