# Supplementary material for: Enhancing reach and effectiveness of recruitment through email: a study within a trial (SWAT)
Source: Trials. 2026 Aug 6;27:643. doi: 10.1186/s13063-026-09902-1 (PMC13729685; doi:10.1186/s13063-026-09902-1)
Supplement: Supplementary file 4 [file 13063_2026_9902_MOESM1_ESM.docx]

**Additional file 1**

**SWAT Reporting Guideline Checklist**

| ***Item No.*** | ***CONSORT 2010 item to be included in publication*** | ***Additional information and example text shown in italics where possible*** | ***Page No.*** |
| --- | --- | --- | --- |
| **Title and Abstract** | | | |
| **1a** | The term ‘SWAT’ should be used in the title | The SWAT registry number should be included if available:  *SWAT [insert number]: [insert title of SWAT]* | 1 |
| **1b** | Structured summary | Structured using these headings: Background, Methods, Results, Conclusion  Details of the host trial(s) included in which the SWAT intervention was evaluated | 2-3 |
| **1c** | Keywords | Include: ‘SWAT’; ‘Study Within A Trial’; the trial process targeted (e.g. ‘recruitment methods’); embedded randomised controlled trial | 3 |
| **Introduction; Background and objectives** | | | |
| **2a** | Scientific background and explanation of rationale for the SWAT | Justify the need for the SWAT; cite systematic review evidence where appropriate  Replication SWAT:  Also cite previous SWAT evaluations undertaken as part of the rationale | 3-6 |
| **2b** | Specific objectives or hypotheses for the SWAT | State SWAT question as objective  *Does [insert SWAT intervention] increase/decrease [outcome] compared to [comparator] in [participants]?* | 6 |
| **Methods; Trial design** | | | |
| **3a** | Description of the SWAT (such as parallel, factorial, cluster), including allocation ratio | Describe the trial design and allocation ratio:  *A [insert number of trial arms and trial design] SWAT was undertaken with an allocation ratio of [insert allocation ratio] (intervention detail vs control detail)*  State where the SWAT protocol is registered:  *The SWAT protocol (number) can be found at [insert details of SWAT repository link]*  If SWAT protocol is not registered, include it as an appendix  Host trial(s):  *The SWAT was embedded in the [insert host trial name(s)]*  Reference the host trial’s registration number(s) and if the protocol(s) for the host trial is/are available elsewhere or include a link to the study project page(s)  Provide a brief description of the host trial(s) using PICO format. At a minimum, age, gender, and ethnicity should be reported per group in addition to any demographics deemed relevant by the host trial team(s); however, we encourage authors to refer to and report in accordance with PROGRESS-PLUS where feasible. If the SWAT was conducted across multiple host trials at the same time, a description of each host trial should be provided  *Host trial Participants; Intervention; Comparator; Outcomes*  State the ethical approval arrangements for the SWAT:  *The SWAT was approved by the Research Ethics Committee [insert name/reference number]*  If changes to the SWAT occurred:  *The following changes occurred once the SWAT started [insert text]* | 6-7 |
| **3b** | State changes (with reasons) to methods of SWAT following commencement |  | 6-9 |
| **Participants** | | | |
| **4a** | State eligibility criteria in SWAT, including differences to those from the host trial(s) | State participant eligibility. This can be tabulated | 7 |
| **4b** | Include setting(s) and location(s) where SWAT data was collected | Describe SWAT data collection methods:  *SWAT data were collected in the following settings/locations [insert text] using the following methods [e.g. face to face, postal follow-up, telephone follow-up, electronic data collection]* | 7 |
| **Interventions** | | | |
| **5** | Describe SWAT intervention to enable replication, including how and when interventions were administered and recruitment dates | Briefly describe the SWAT intervention and control. Reference to the SWAT protocol for further details is acceptable if the protocol is available to the reader | 7-9 |
| **Outcomes** | | | |
| **6a** | State primary and secondary outcome measures for the SWAT  Include how and when they were assessed | State the primary and outcome measures for the SWAT:  *Primary outcome measure: [insert information including how/when/who assessed]*  *Secondary outcome measure(s): [insert information including how/when/who assessed]*  This information can be tabulated  If appropriate:  *The following changes occurred once the SWAT started [insert text]* | 9 |
| **6b** | Include changes (and reasons) to SWAT outcomes after commencement |  | N/A |
| **Sample size** | | | |
| **7a** | How sample size was determined for the SWAT | SWATs are often individually underpowered due to the sample size being constrained by the host trial(s). A robust estimate of the effect of the SWAT intervention might therefore depend on the aggregation of replicated SWAT evaluations. It is not expected that a formal sample size calculation will always be done  *The SWAT sample size depended on the host trial(s) [insert host trial name]; therefore no formal sample size calculation was performed, which is in line with SWAT methodology*. [insert any reasoning for a subsample of the host trial(s) being used – e.g. SWAT was included midway through the trial]  State if interim analyses and/or stopping rules were planned or not  If interim analyses and/or stopping rules were planned:  *The following interim analyses were planned [state analyses here]. The stopping rules were [details here]* | 9 |
| **7b** | When applicable, explanation of any interim analyses and stopping rules for the SWAT |  | N/A |
| **Randomisation: Sequence generation** | | | |
| **8a** | The method used to generate the random allocation sequence for the SWAT | Provide details of the method of randomisation:  *Participants were randomised by [insert method with all methodological details]* | N/A |
| **8b** | Type of randomisation; details of any restriction (such as blocking and block size) |  | N/A |
| **Allocation concealment mechanism** | | | |
| **9** | The mechanism used to implement the random allocation sequence (such as sequentially numbered containers), describing any steps taken to conceal the sequence until interventions were assigned for the SWAT | Provide details of the method of allocation concealment:  *Allocation concealment was achieved by [insert method]* | N/A |
| **Implementation** | | | |
| **10** | Who generated the random allocation sequence, who enrolled participants, and who assigned participants to interventions for the SWAT | Provide details of randomisation sequence generation and implementation:  *Randomisation was performed by [specify centre or personnel], [specify centre or personnel] enrolled participants and [specify centre or personnel] assigned the participant to the SWAT intervention or comparator* | N/A |
| **Blinding** | | | |
| **11a** | If done, who was blinded after assignment to the SWAT interventions (for example, participants, care providers, those assessing outcomes), and how | Explain who was blinded and if individuals were not blinded note the implications of this: *The [specify stakeholder group, e.g. participants, SWAT team members, outcome assessors, statisticians] were blind and the [specify stakeholder group, e.g. participants, SWAT team members, outcome assessors, statisticians] were not blind to the SWAT intervention.[Note implications of unblinded stakeholders as relevant]* | N/A |
| **11b** | If relevant, a description of the similarity of the SWAT interventions |  | N/A |
| **Statistical methods** | | | |
| **12a** | Statistical methods used to compare groups for primary and secondary outcomes for the SWAT | All analyses for the SWAT should be preplanned, ideally detailed in a SWAT Statistical Analysis Plan (SAP), which might be a short component of the SWAT registry entry. Unless detailed thoroughly and extensively in a publicly available SWAT protocol, the analysis for each outcome should be detailed in the methods of the report. Alternatively, the SAP could be uploaded as supplementary material depending on the journal  The analysis section should include the software used, the statistical methods (including significance level for hypothesis testing), and the population used for the analysis (e.g. intention-to-treat or per-protocol) | 10-11 |
| **12b** | Methods for additional analyses, such as subgroup analyses and adjusted analyses |  | 10-11 |
| **Results** | | | |
| **Participation flow** | | | |
| **13a** | For each group, the numbers of participants who were randomly assigned, received intended SWAT intervention, and were analysed for the primary outcome of the SWAT | Provide a participant flow diagram that includes this data  Include details of the host trial(s) participants excluded from the SWAT, with reasons, where appropriate | 11 |
| **13b** | For each group participating in the SWAT, losses, and exclusions after randomisation, together with reasons |  | N/A |
| **Baseline data** | | | |
| **15** | A table showing baseline demographic and clinical characteristics for each group | The context of the host trial(s) for each SWAT evaluation is likely to be different and contextual information about the host trial(s) should be provided  In addition to general information about the host trial(s) (see ‘Methods’), we suggest a table of participant baseline characteristics for those allocated to each group of the SWAT evaluation if these details are available. At a minimum, age, gender, and ethnicity should be reported per group in addition to any demographics deemed relevant by the host trial team, however, we encourage authors to refer to and report in accordance with PROGRESS-PLUS where feasible | 11-12 |
| **Numbers analyzed** | | | |
| **16** | For each group of the SWAT, the number of participants (denominator) included in each analysis and whether the analysis was by originally assigned groups |  | 8, 11 |
| **Outcomes and estimation** | | | |
| **17a** | For each primary and secondary outcome, results for each group, and the estimated effect size and its precision (such as 95% confidence interval) | Results should be presented in tables as far as possible rather than only being presented in the body of the text. To facilitate meta-analysis, SWATs should report the actual number of participants in each group in the SWAT evaluation  A key element of SWAT evidence is their ability to be replicated. An important principle for reporting research is that new findings should be placed in the context of existing, relevant evidence. Therefore, we recommend, where possible, that an updated meta-analysis be included that presents the results of the current SWAT combined with previous evaluations of the SWAT intervention. Presentation as a cumulative meta-analysis is particularly helpful because it would help to inform judgements about the need for further evaluations of a SWAT intervention | 12-16 |
| **17b** | For binary outcomes, the presentation of both absolute and relative effect sizes is recommended |  |  |
| **17c** | Costs associated with the SWAT | Summarise the costs associated with the SWAT:  *The total cost of the SWAT was [insert cost], which equates to [insert cost] per participant*  Tabulate the additional costs to the trial incurred because of the SWAT, including total cost and cost per participant. This may include direct costs (e.g. printing, postage, animation) and indirect costs (e.g. staff time to prepare mailings). As SWAT evaluations generally need replication, it is useful for trialists to see the costs of both using the SWAT intervention *and* the cost of evaluating the SWAT should they wish to replicate the evaluation  If a positive effect (irrespective of statistical significance) was identified, provide a cost per additional participant for whom there is a favourable result (e.g. cost per participant retained). Otherwise, note that cost per participant was not derived | N/A |
| **Ancillary analyses** | | | |
| **18** | Results of any other analyses performed on the SWAT data, including subgroup analyses and adjusted analyses, distinguishing pre-specified from exploratory |  | 16 |
| **Harm** | | | |
| **19** | All important harm or unintended effects in each group that took part in the SWAT (for specific guidance, see CONSORT for harm) | If no harm or unintended effects were collected, this should also be noted | N/A |
| **Discussion** | | | |
| **20** | Interpretation consistent with results, balancing benefits and harm, and considering other relevant evidence | Within the discussion, reflect on the population demographics in the context of equality, diversity, and inclusion (e.g. Does the SWAT population reflect the host trial population(s)? If not, why not?) | 17-21 |
| **Limitations** | | | |
| **21** | SWAT limitations, addressing sources of potential bias, imprecision, and, if relevant, multiplicity of analyses for the SWAT |  | 17-21 |
| **Generalisability** | | | |
| **22** | Generalisability (external validity, applicability) of the SWAT findings |  | 17-21 |
| **Implications** | | | |
| **23** | Implications for trial practice and SWAT research | These could make use of the cumulative meta-analysis and Trial Forge Guidance 2 on whether further evaluations of the intervention are warranted  Consideration should be given to any other replications of the same SWAT and whether the findings are consistent with these or not. In addition, consideration should be given to the populations of other replications of the same SWAT when considering future SWAT research | 17-21 |
| **Other information** | | | |
| **24** | Registration  Registration number and name of trial registry | Include the information for both the host trial(s) and SWAT  It is recommended that SWATs are registered on a repository to ensure all SWATs performed can be included in the evidence base and support future replication  The following repository is available to register SWATs: the Northern Ireland Methodology Hub’s SWAT repository (this repository is for SWATs and encourages replications of registered SWATs): [https://www.qub.ac.uk/sites/ TheNorthernIrelandNetworkforTrialsMethodology %E2%80%8C%E2%80% 8CResearch/SWATSWARInformation/ Repositories/SWATStore/](https://www.qub.ac.uk/sites/%20TheNorthernIrelandNetworkforTrialsMethodology%20%E2%80%8C%E2%80%25%208CResearch/SWATSWARInformation/%20Repositories/SWATStore/)  SWATs may also be included in the ISRCTN trial registry (<https://www.isrctn.com/>) and/or the Clinical Trials database (<https://clinicaltrials.gov/>)  as part of the host trial(s) | 6 |
| **25** | Protocol  Where the full trial protocol can be accessed, if available | Include the information for both the host trial(s) and SWAT | N/A |
| **26** | Funding  Sources of funding and other support (such as supply of drugs), role of funders | Include the information for both the host trial(s) and SWAT | 6 |
| **Additional** | | | |
|  | Data sharing | We suggest authors make the data used to generate their results available as a supplementary file or through data-sharing platforms such as OSF (<https://osf.io> ) | N/A |

Citation:

Arundel CE, Clark LK, Parker A, Beard D, Coleman E, Cooper C, et al. Trial Forge Guidance 4: a guideline for reporting the results of randomised Studies Within A Trial (SWATs). Trials. 2024 Mar 12;25(1):183.
